# Extensive Subcutaneous Emphysema Post-traumatic Tube Thoracostomy

**DOI:** 10.7759/cureus.34428

**Published:** 2023-01-31

**Authors:** Mariam Mallat, Abdallah Diab, Jerar Bleibel, Layal Olaywan

**Affiliations:** 1 Department of Pulmonary and Critical Care Medicine, Lebanese University, Faculty of Medical Sciences, Beirut, LBN; 2 Department of Pulmonary and Critical Care Medicine, Lebanese Hospital Geitaoui - University Medical Center, Beirut, LBN

**Keywords:** thoracic radiology, broncho cutaneous fistula, subcutaneous emphysema, lung injury, traumatic tube thoracostomy

## Abstract

Bronchocutaneous fistula (BCF) is a pathologic communication between the bronchus and the subcutaneous tissue. Its diagnosis is made mainly by chest imaging, and bronchoscopy can help in accurately localizing the fistula. Treatment options include conservative and non-conservative approaches. We report a case of iatrogenic bronchocutaneous fistula occurring after traumatic chest tube placement in an 81-year-old man, treated efficiently with conservative management.

## Introduction

Tube thoracostomy is an invasive procedure commonly performed to treat a variety of pleural diseases, with a complication rate of up to 30% [1]. Bronchocutaneous fistula is a rarely reported complication of tube thoracostomy caused by intraparenchymal tube placement. We describe the case of an 81-year-old man who was complicated by a bronchocutaneous fistula after traumatic chest tube insertion.

## Case presentation

An 81-year-old man, smoker, with a history of hypertension, coronary artery disease, and heart failure with an estimated ejection fraction of 30%, was referred to our institution post right thoracic chest tube placement for preemptive diagnosis of right-sided pneumothorax, based on clinical examination.

The patient’s history goes back to one day prior to the presentation, when he had right-sided chest trauma after falling on the ground. Then, he was only experiencing pain without dyspnea or hemoptysis. Several hours later, he started to have swelling in the right chest wall.

The next day, he presented himself to the nearest emergency department. As mentioned by his daughter, he was not in respiratory distress, and he was saturating well without supplemental oxygen. On the spot, the emergency physician inserted a chest tube into his right chest cavity after noticing subcutaneous emphysema on physical examination without doing any diagnostic chest imaging.

A chest computed tomography (CT) performed thereafter confirmed intraparenchymal chest tube placement into the right lung, with mild surrounding subcutaneous emphysema and multiple right rib fractures associated with a minimal right pneumothorax.

**Figure 1:**
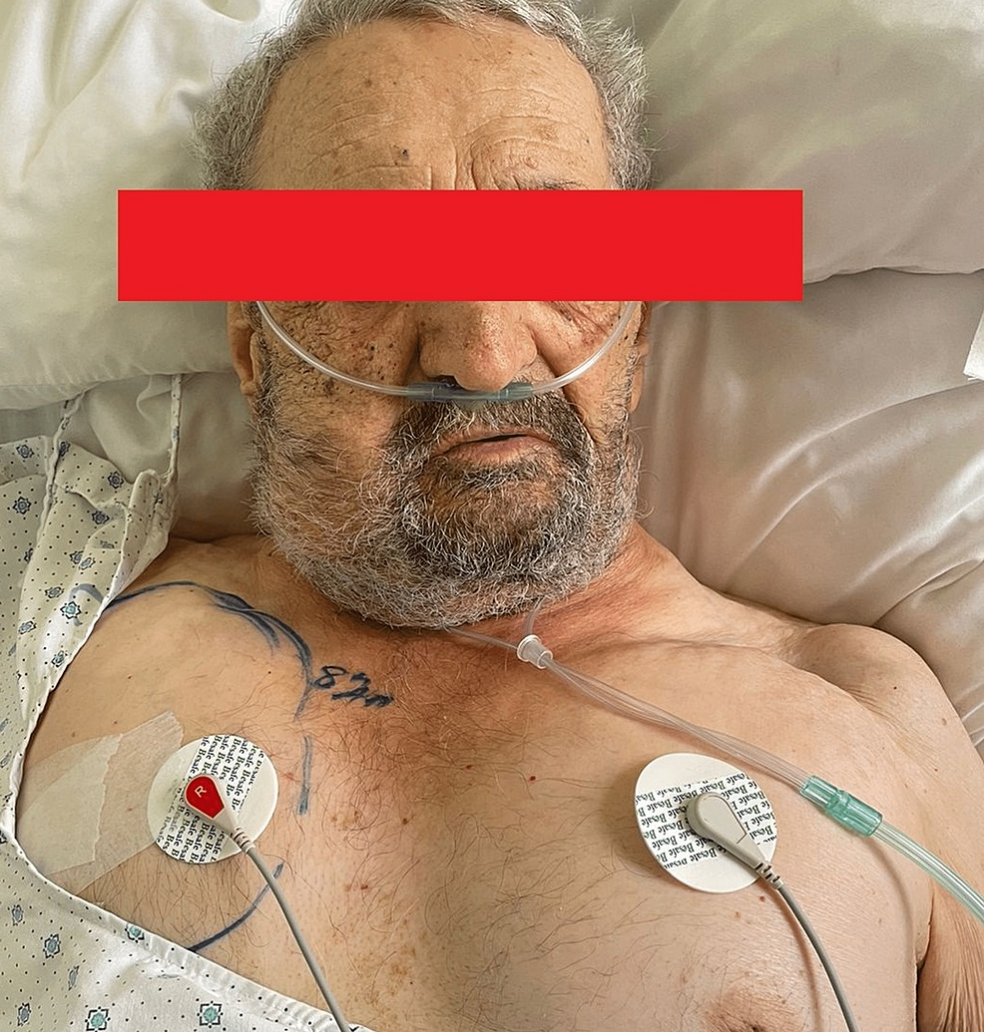
Bilateral chest subcutaneous emphysema extending to the neck.

Next, the patient was transported to our hospital. The chest tube was accidentally removed during transport. On arrival, his vital signs were stable. On physical examination, he was breathing comfortably, but there was a notable increase in the edema causing swelling on the face that prevented the eyes from completely opening, as well as swelling of the neck and chest bilaterally (Figure 1) with crepitus on palpitation and a decrease in breath sounds bilaterally.

**Figure 2:**
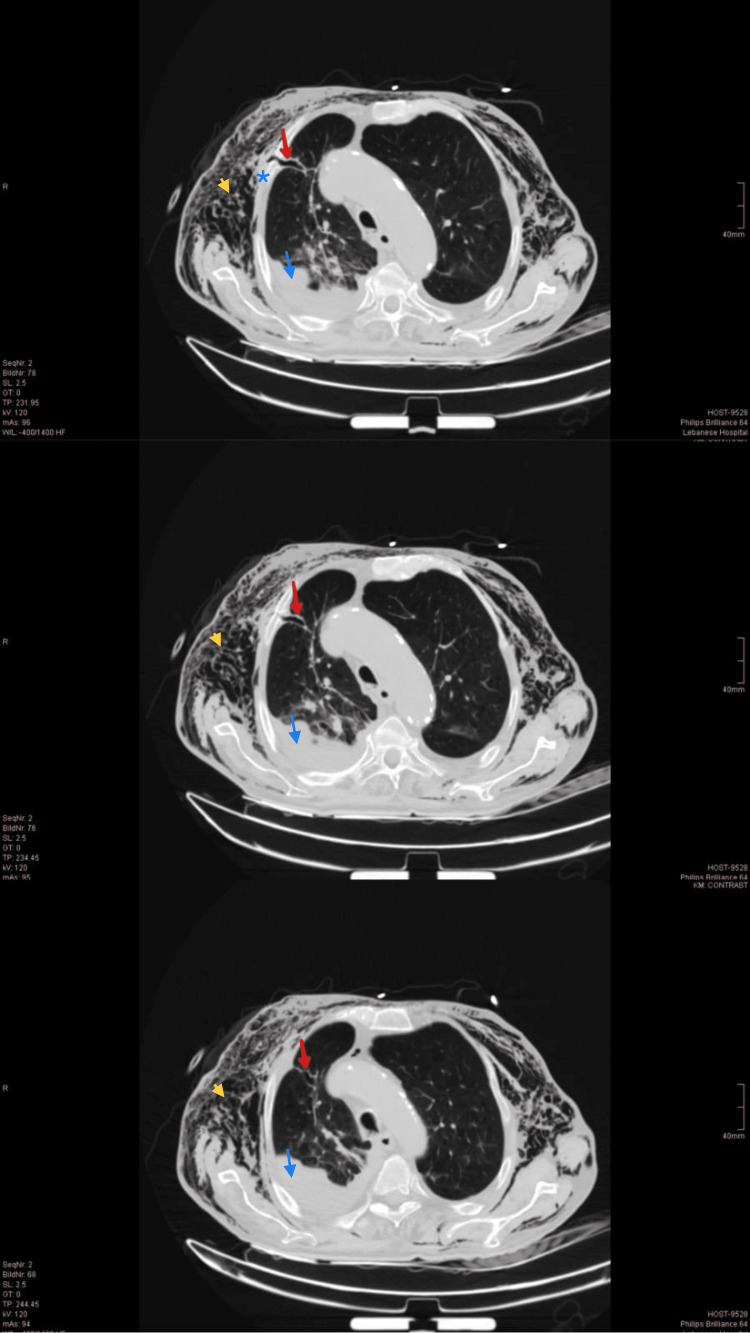
Bilateral severe subcutaneous emphysema (yellow arrowheads). Fistulous tract at the site of earlier chest tube (red arrows) in communication with the subcutaneous tissues. Right lower lobe collapse/consolidation (blue arrows). Rib fracture (blue asterisk).

A repeated chest CT scan (Figure 2) revealed a fistulous tract in the right lung parenchyma communicating with the subcutaneous tissues and delineating the passage site of the retracted chest tube, surrounded by exuberant emphysema extending from the chest to the neck with no evidence of pneumothorax.

Given the patient’s cardiac comorbidities, which classified him as high-risk for any invasive intervention, a bronchoscopy for a definitive localization of a BCF was not performed. Moreover, there was a consensus to adopt a conservative therapeutic approach since his clinical status was relatively stable. Thus, he was admitted to the critical care unit to be closely monitored for any respiratory or hemodynamic compromise. Fortunately, the subcutaneous emphysema decreased significantly over a period of 72 hours and almost resolved after two weeks without causing any airway compromise despite being extensive and severe. The patient was discharged home in good condition, but a follow-up chest imaging was not accomplished due to loss of contact.

## Discussion

A bronchocutaneous fistula is described as an unhealed tract communicating the bronchus and the (sub-)cutaneous part of the chest wall through a break in the parietal pleura [2]. It is a rare complication following tube thoracostomy [2], pneumonectomy [3], positive pressure ventilation [4], infection with Mycobacterium tuberculosis [5], chronic calcified empyema [6], pleural squamous cell carcinomas [7], etc. Interestingly, three examples of postmortem pulmonary perforations, clinically unknown before death, were described in a case series as complications of chest tube insertion [8].

The identification of BCF may represent a major challenge. Physical examination has a role in the primary diagnosis of BCF, as the Valsalva maneuver results in a high-pitched squeak sound over the affected area of the chest [9]. Simple chest radiography may show air in the subcutaneous space [10]. Whereas, CT chest visualizes the fistulous tract from the bronchus to the chest wall, making it a tool for a definitive diagnosis of BCF [2]. Bronchoscopy with dye instillation, such as methylene blue, represents a useful method to accurately diagnose and localize the fistula opening in the bronchial tree [11], allowing the bronchoscopist to treat it properly.

In regard to our patient, a diagnostic bronchoscopy was not undertaken given his comorbidities and the extensive subcutaneous emphysema extending to the neck and face, which could precipitate a respiratory compromise at any time. Strong evidence of a BCF was delineated by the remarkable majorization of subcutaneous emphysema after chest tube removal and the presence of an intraparenchymal fistulous tract communicating with the subcutaneous tissues. A possible explanation corroborating the clinical picture could be sustained trauma to the subsegmental bronchi at the time of chest tube insertion, which was responsible for the fistula formation and subsequent air leak to the subcutaneous tissues.

Moreover, we believe that the insertion of the chest tube in a stable patient, relying only on the clinical judgment of the physician, who had not performed any diagnostic chest imaging to verify the size of the pneumothorax, resulted in a direct lung injury and BCF formation.

Therapeutic approaches include thoracoscopic repair with a seromuscular flap [12] or bronchoscopic fixation via endobronchial valves [13], histo-acryl glue [14], or stenting [15] to seal the leak once the location of the sinus tract is accurately localized. Chemical pleurodesis represents another treatment option [16]. Talc poudrage or tetracycline derivatives can be used. Sometimes physicians opt for conservative management [17], depending on the causative agent and the patient’s clinical status and preferences, as chosen by the patient.

## Conclusions

Chest imaging before chest tube placement in stable patients identifies the extent of the pneumothorax and the indication for drainage. BCF is one of the rare complications following traumatic chest tube insertion. CT chest and bronchoscopy may be essential for definitive diagnosis and accurate fistula localization, respectively. The optimal therapeutic approach is not yet defined. Thus, adopting conservative management may be a reasonable option, especially for patients who are not considered candidates for invasive interventions.
